# Immunosenescence, gut dysbiosis, and chronic kidney disease: Interplay and implications for clinical management

**DOI:** 10.1016/j.bj.2023.100638

**Published:** 2023-07-29

**Authors:** Tao Han Lee, Jia-Jin Chen, Chao-Yi Wu, Ting-Yun Lin, Szu-Chun Hung, Huang-Yu Yang

**Affiliations:** a Nephrology Department, Chansn Hospital, Taoyuan, Taiwan; b Kidney Research Center, Nephrology Department, Chang Gung Memorial Hospital in Linkou, Chang Gung University College of Medicine, Taoyuan, Taiwan; c Division of Allergy, Asthma, And Rheumatology, Department of Pediatrics, Chang Gung Memorial Hospital, College of Medicine, Chang Gung University, Taoyuan, Taiwan; d Division of Nephrology, Taipei Buddhist Tzu Chi General Hospital, Buddhist Tzu Chi University, Taipei, Taiwan; e Department of Health Policy and Management, Johns Hopkins Bloomberg School of Public Health, Baltimore, MD, USA

**Keywords:** immunosenescence, inflamm-aging, CKD, gut dysbiosis

## Abstract

Immunosenescence refers to the immune system changes observed in individuals over 50 years old, characterized by diminished immune response and chronic inflammation. Recent investigations have highlighted similar immune alterations in patients with reduced kidney function. The immune system and kidney function have been found to be closely interconnected. Studies have shown that as kidney function declines, both innate and adaptive immunity are affected. Chronic kidney disease (CKD) patients exhibit decreased levels of naive and regular T cells, as well as naive and memory B cells, while memory T cell counts increase. Furthermore, research suggests that CKD and end-stage kidney disease (ESKD) patients experience early thymic dysfunction and heightened homeostatic proliferation of naive T cells. In addition to reduced thymic T cell production, CKD patients display shorter telomeres in both CD4+ and CD8+ T cells.

Declining kidney function induces uremic conditions, which alter the intestinal metabolic environment and promote pathogen overgrowth while reducing diversity. This dysbiosis-driven imbalance in the gut microbiota can result in elevated production of uremic toxins, which, in turn, enter the systemic circulation due to compromised gut barrier function under uremic conditions. The accumulation of gut-derived uremic toxins exacerbates local and systemic kidney inflammation. Immune-mediated kidney damage occurs due to the activation of immune cells in the intestine as a consequence of dysbiosis, leading to the production of cytokines and soluble urokinase-type plasminogen activator receptor (suPAR), thereby contributing to kidney inflammation.

In this review, we delve into the fundamental mechanisms of immunosenescence in CKD, encompassing alterations in adaptive immunity, gut dysbiosis, and an overview of the clinical findings pertaining to immunosenescence.

**At a glance commentary**

**Scientific background on the subject**

Immunosenescence, which becomes apparent in individuals aged 50 and above, involves the manifestation of diminished immune responses and chronic inflammation. This phenomenon is also observed in patients with reduced kidney function. The growing recognition of the interconnectedness between immunity and kidney health underscores the importance of this field.

**What this study adds to the field**

In the present review, we delve into the fundamental mechanisms underlying immunosenescence within chronic kidney disease (CKD). This examination comprehensively explores alterations in adaptive immunity, gut dysbiosis, and clinical observations. By unveiling the intricate interplay between immune decline and kidney function, this review sheds light on the complex relationships. These insights pave the way for potential therapeutic strategies targeting both immune response and kidney inflammation.

## Introduction

Immunosenescence usually refers to the phenomenon of defective immune response and chronic inflammation caused by changes in the immune system in healthy individuals aged 50 years or older. As individuals age, the process of autophagy in immune cells declines, impeding the breakdown of dysfunctional mitochondria. This results in the accumulation of reactive oxygen species and DNA damage. These physiological changes, along with aging, impair vaccine responses and increase susceptibility to infection, autoimmunity, and cancer [1].

Chronic kidney disease (CKD) is typically defined as kidney damage or a glomerular filtration rate (GFR) of <60 mL/min/1.73 m^2^ or proteinuria/structural change for 3 months or more [2]. Previous studies have shown that as kidney function declines, CKD patients are predisposed to a higher cardiovascular risk, increased susceptibility to infection and certain types of cancers, and reduced response to immunization [3,4]. According to a study based on the US Renal Data System, from 2001 to 2010, the mortality risk due to sepsis is approximately 250-fold higher among end stage kidney disease (ESKD, also known as kidney failure) patients than in the general population [5]. An international collaborative study revealed a significantly increased risk of certain cancers in patients under dialysis. This study reported that ESKD patients had a 1.6–4 standardized incidence ratio (SIR) of cervical cancer, which is usually secondary to papillomavirus, and a 1.2–1.5 SIR of liver cancer, which may be associated with hepatitis B and C viruses [6].

Currently, investigators have revealed the close and interactive connection between the immune system and kidney function, which is independent of the underlying disease of kidney insufficiency and may be one reason why patients with kidney insufficiency are more fragile. Previous studies have grouped CKD patients into the “inflamm-aging” condition, which shifts lymphocytes toward a senescent and exhausted phenotype, similar to the aging process [7,8]. Immunological aging in patients with ESKD is accelerated and increased by 20 years compared with age-matched healthy individuals [1]. Furthermore, kidney insufficiency alters the environment inside the intestinal lumen, leading to gut dysbiosis, which further affects intestinal immunity. Moreover, several factors have been observed to be associated with heightened systemic inflammation during the dialysis procedure. These include the transmembrane passage of bacterial lipopolysaccharide (LPS) from contaminated dialysate into the blood component [9], activation of mononuclear cells and the subsequent increase in production of proinflammatory cytokines upon contact with the dialysis membrane [10], activation of the complement system upon blood–membrane interaction [11], and an increase in reactive oxygen species (ROS) and depletion of antioxidants during hemodialysis [12]. In this review, we discuss the key mechanisms of immunosenescence in CKD, from changes in adaptive immunity to gut dysbiosis, and summarize the clinical results of immunosenescence.

## Altered innate and adaptive immunity in chronic kidney disease

### Change of innate immunity in chronic kidney disease

The innate immunity is characterized by non-specific recognition of self and non-self stimuli through pattern recognition receptors (PRRs). These receptors are present in various myeloid cells, which serve as the first line of defense against pathogens. Activation of PRRs triggers immune cell activation, complement system activation, and coagulation system activation [13,14]. Multiple studies have reported increased expression of PRRs in different kidney diseases. For instance, TLR4 (TLR, toll-like receptor) expression is increased in lupus nephritis [15], antineutrophil cytoplasmic antibody-associated vasculitis/nephritis [16], sepsis-associated kidney injury [17], ESKD [18], and aging kidney [19]. Xi et al. demonstrated through animal models that several components of innate immunity are overexpressed, including TLR1, 2, 3, 4, 5, 11, as well as heat-shock protein 70 (HSP70) and high mobility group box 1 (HMGB1). It is proposed that TLR upregulation leads to the induction of proinflammatory cytokines, fibrosis, and progression of CKD [19]. According to this investigation, different kidney diseases or aging kidneys can activate innate immunity in the kidney, resulting in the progression of CKD [19].

Kidney disease can lead to increased local innate immunity in the kidney, as well as systemic activation and dysfunction of the innate immune system in various aspects [20]. Patients with ESKD exhibit expansion of the CD14+CD16+ subset in circulating monocytes, along with increased expression and activity of TLR4 in neutrophils and monocytes [18]. The production of pro-inflammatory cytokines tumor necrosis factor (TNF) and interleukin-6 (IL-6) by activated monocytes was also increased [18], which was associated with increased systemic inflammation. However, impaired phagocytic activity against pathogens has been observed in uremic patients [21]. Uremia is also associated with depletion of the plasmacytoid dendritic cell subset, and the hemodialysis procedure significantly impairs the ability to produce tumor necrosis factor-α (TNF-α) in response to LPS [22]. Verkade et al. demonstrated impaired stimulation of allogeneic T-cells by dendritic cells in advanced kidney disease patients [23]. For short, uremia is associated with dysregulation of innate immunity, characterized by upregulation of chemokine/chemokine and ROS production [24], impaired phagocytosis, and impaired stimulation of T cells [20].

### Change of adaptive immunity in chronic kidney disease

The adaptive immune system generates immunological memory following an initial response to a specific pathogen, which can lead to an experienced and enhanced response when re-encountering that pathogen in the future. In patients with declining kidney function, nitrogenous compounds and cytokines that are usually cleared by the kidney accumulate in the body, a condition known as uremia. Uremia has a detrimental effect on the quantity and quality of the adaptive immune system [3]. The number of naive T cells, regular T cells, and both naive and memory B cells decrease, while memory T cells increase in number. By analyzing the profile of T cell subsets, Litjens et al. observed a decrease of 50% in naive CD4 T cells (CCR7+CD45RO-) and a corresponding 34% rise in central memory CD4 T cells (CCR7+CD45RO+) among CKD stage 5 patients as compared to healthy individuals. A similar cellular shift was also observed in the group of CD8 T cells, with a reduction in naive CD8 T cells (CCR7+CD45RO-) and an increase in effector memory CD8 T cells (CCR7-CD45RO-). Litjens and colleagues attributed the change in the composition of T cell subsets in CKD patients to impaired cytokine production, especially interleukin-17 (IL-17) [25]. Similar conclusions were drawn by Yoon et al. [26].

**Fig. 1:**
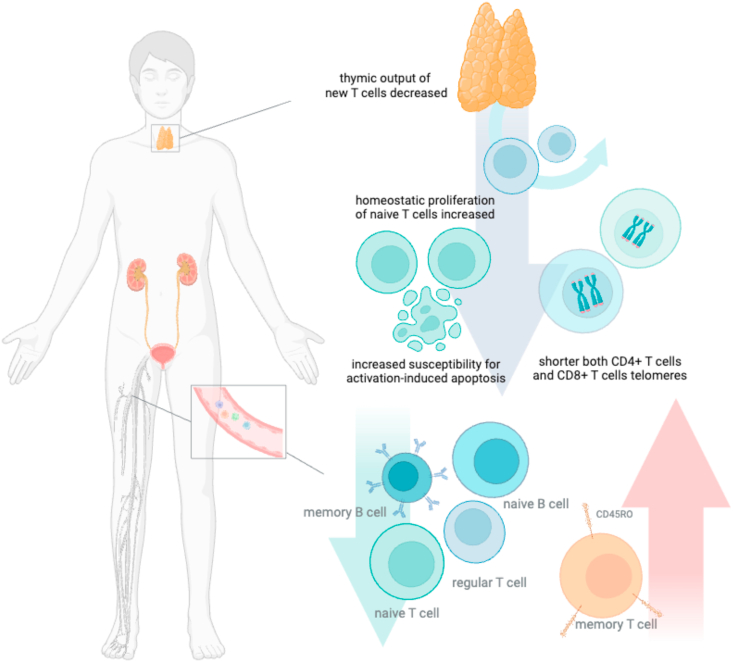
The shift of lymphocytes towards a senescent and exhausted phenotype, as well as a premature decline in thymic function, in patients with chronic kidney disease (CKD).

Not only did investigators reveal the shift in CD4 and CD8 T cell composition, but they also reported that CKD and ESKD patients showed similar phenomena to the aging population: premature decline in thymic function. After reaching 40 years of age, the thymic output of new T cells (recent thymic emigrants) in the bloodstream significantly diminishes, and the maintenance of T-cell levels in circulation is sustained by homeostatic proliferation [27]. Betjes et al. investigated ESKD patients and found that they had a decreased number of recent thymic emigrants and increased homeostatic proliferation of naive T cells. They also revealed that with increased homeostatic proliferation, naive T cells in ESKD patients are more susceptible to activation-induced apoptosis [28]. It is worth noting that thymic T cell output was significantly lower in the CKD stage 5 population compared with the healthy, age-related population, with shorter T cell telomeres in both CD4+ and CD8+ T cells [Fig. 1] [29]. Meijers et al. also confirmed the reduction of CD28 expression in CD4 and CD8 subpopulations, which implies that both CD4 and CD8 groups of cells tend to be more differentiated in patients with poor kidney function [29]. Kidney replacement therapy modalities do not affect the immunosenescence phenomenon in the CKD population. In a comparison of immunological changes between ESKD patients undergoing hemodialysis and peritoneal dialysis, no significant variations in T cell counts and aging indicators were observed, indicating that immunosenescence is a universal occurrence in ESKD patients, irrespective of the treatment approach [29,30].

Although several studies have demonstrated an association between the upregulation of T follicular helper cells (T_FH_ cells) and chronic inflammation in cardiovascular and autoimmune diseases, Hartzell and colleagues reported no difference in total T_FH_ (CXCR5+PD1+CD4+CD8−) levels among patients with ESKD, CKD and the healthy population. However, ESKD patients had significantly lower frequencies of T_FH1_ (CCR6−CXCR3+CXCR5+PD1+CD4+CD8−) cells but more T_FH2_ (CCR6−CXCR3−CXCR5+PD1+CD4+CD8−) and T_FH17_ (CCR6+CXCR3−CXCR5+PD1+CD4+CD8−) cells. A previous study suggested that T_FH2_ contributes to B cell maturation by aggravated B cells converting into plasma blasts in germinal centers [31], which may explain the significant increase in circulating plasmablasts observed in ESRD patients in the same study [32].

It is worth noting that uremic status also decreases TLR expression, and one of TLR's function is involved in the maturation of dendritic cells [3,33,34]. Dendritic cells, as one of the antigen-presenting cells (APC), primarily function to present antigens to lymphocytes and induce their activation. By lowering TLR expression, lymphocyte activation is reduced in infection-prone CKD and ESKD patients, which increases their susceptibility to bacterial infections.

Uremic status not only induces lymphocyte phenotype shifting and increased differentiation status, but also affects inflammatory cytokine patterns. In 2020, Hartzell and colleagues analyzed the inflammatory cytokine patterns of ESRD, CKD patients, and the healthy population, revealing that ESKD patients had significantly higher serum levels of interferon-gamma (IFN-γ), TNF-α, soluble CD40L (sCD40L), Granulocyte-macrophage colony-stimulating factor (GM-CSF), interleukin-4 (IL-4), interleukin-8 (IL-8), Monocyte chemoattractant protein-1 (MCP-1), and Macrophage inflammatory protein-1β (MIP-1β) than CKD and the healthy population. Following mitogen stimulation, CD4+ and CD8+ T cells in the ESRD group exhibited a pro-inflammatory phenotype with heightened levels of IFN-γ and TNF-α. On the other hand, both CKD and ESKD patients had elevated interleukin-2 (IL-2) levels [32]. Al-rawi et al. further reported a significant increase in IL-2 and IL-17 levels, which were paralleled with an increase in serum creatinine, urea, and urinary albumin concentration in 2022 [35].

Uremia-associated dysregulation of APCs, T cells, and cytokine expression can lead to impaired vaccine responses [36,37], increased proinflammatory status [38], and a higher risk for infection [33] and cardiovascular events [39,40]. Decreased B cell activation and increased apoptosis can also diminish serologic responses against pathogens and immunogenicity after vaccination [3]. In summary, CKD and ESKD are associated with adaptive immunosenescence, which is manifested as a decreased capacity of T and B cells against new infections, decreased naive lymphocytes, increased differentiated lymphocyte strain, and increased inflammatory cytokines.

## Chronic kidney disease induces gut dysbiosis

The gut is the largest immune organ in the body, with a complex mucosal immune system. Intestinal epithelial cells directly participate in immunological surveillance and direction of host responses in the gut, and lymphocytes and innate immune cells are found throughout the epithelial layers [41]. Recent studies have revealed that not only does the complex immune system of the gut play an important role in human physiology and pathophysiology, but the trillions of microorganisms harbored by the gut also host a special role in physiological and immunological protection. Nowadays, gut microbiota is considered the bridge between aging, immune system, and kidneys. It plays a role in organ cross-talk via the gut–kidney axis and the gut–brain axis through immune regulation [42]. Gut microbiota could activate the bone marrow-derived immune cells, which could result in low-grade inflammation [42]. Yang et al. demonstrated the significant role of gut microbiota in bone formation and health, highlighting their ability to induce insulin-like growth factor-1 (IGF-1), which promotes bone formation [43]. They observed higher expression of IGF-1 in the bone marrow of mice colonized with microbiota compared to germ-free mice. Additionally, short-term colonization of microbiota led to increased expression of nuclear factor-kappaB ligand (RANKL), TNF-α, and interleukin-1 beta (IL-1β) in the colon, as well as increased RANKL expression in the bone marrow [43]. Another study by Josefsdottir et al. reported that depletion of the intestinal microbiome through broad-spectrum antibiotics resulted in the depletion of hematopoietic stem cells and impaired granulocyte maturation, which could be partially reversed by colon fecal microbiota transplantation [44]. Thus, both studies by Yang and Josefsdottir suggest that the gut microbiome plays a crucial role in bone formation, bone health, and the activity of bone marrow hematopoietic stem cells through hormone and cytokine production. In the CKD and ESKD population, several factors contribute to gut dysbiosis and increased intestinal permeability. These factors include metabolic acidosis, uremic toxins, congestion of the intestinal wall, vascular calcification associated with bowel ischemia, and frequent exposure to antibiotics [45]. The CKD diet also contributes to alterations in gut microbiota [45,46]. Animal models, as demonstrated by Andersen et al. have shown that uremia is associated with intestinal barrier disruption and translocation of intestinal bacteria, leading to increased systemic inflammation [47], which is consistent with the findings reported by Konrad [48] and Yang [49]. For short, uremia in CKD disrupts the composition of the colon microbiota and epithelial junction, leading to bacteria translocation, endotoxemia, and systemic inflammation. The translocation of bacteria has been demonstrated in early uremic animal models through direct culture methods [50], as well as through the detection of bacterial DNA from feces and circulation in animal model and patient with kidney function declined or on dialysis [[51], [52], [53]]. These phenomena have been proposed as leaky gut syndrome in CKD [45,54]. Although not in patients with declined kidney function, a recently published study by Bernard-Raichon provided some evidence linked between gut dysbiosis and bacteremia. By analyzing the blood culture result, they investigated paired microbiome data between gut microbiome dysbiosis and microbial bloodstream infections indicating that bacteria may translocate from the gut into the systemic circulation in coronavirus disease 2019 (COVID-19) patients [55].

Similar alterations in the adaptive immune system are observed in both the aging population and individuals with kidney failure. Additionally, these two populations also exhibit comparable changes in their gut environment. Age-related gut microbiota changes, including decreasing diversity and expansion in proteolytic bacteria, were reported in a previous study [56]. Furthermore, old age also resulted in increased gut permeability. By investigating the biomarker of plasma intestinal permeability, zonulin, previous studies have demonstrated the disruption of the gut-blood barrier in aging individuals. These studies have reported that leaky gut syndrome may play a role in the age-related increase in inflammation and frailty [[57], [58], [59]]. With declining kidney function, the intestinal environment was modulated. Uremia influences metabolic derangements and promotes pathogen overgrowth. In 2012, Wang and colleagues reported that all the observed genera, including *Klebsiella* spp, *Proteus* spp, *Escherichia* spp, *Enterobacter* spp, and *Pseudomonas* spp, were overgrown in the guts of ESKD patients, and bacterial DNAs can be detected in the blood of 20% of ESKD patients [51]. Besides the overgrowth of microbiota, the change in diversity and enterotype shift was also observed in CKD and ESKD patients. Jiang et al. reported that the enterotypes change from *Prevotella* to *Bacteroides* in ESRD patients compared to the healthy individual [60]. Vaziri and colleagues analyzed the stools from ESKD patients and revealed that kidney failure status significantly modifies the composition of the gut microbiome. There is a decrease in the *Lactobacillus* family and an increase in the *Enterobacteriaceae* family under uremic status [61]. Hu et al. also revealed that patients with CKD exhibited a distinct gut microbial community compared to the healthy population. CKD patients displayed lower abundances of *Candida*, *Bjerkandera*, *Rhodotorula*, and *Ganoderma*, while exhibiting a higher abundance of *Saccharomyces* [62]. Asgharian and colleagues elucidated that alterations in the gut microbiome may contribute to uremic toxicity and inflammation in individuals with ESKD. They discovered a significant positive correlation between the *Prevotellaceae* family and total antioxidant capacity, *Lactobacilli* species and C-reactive protein as well as p-cresol, and *Scardovia wiggsiae* and indoxyl sulfate (IS) [63]. In the review authored by Huang et al. it was summarized that in CKD patients, there is a noteworthy decrease in the levels of *Lactobacillus*, *Bifidobacterium*, *Bacteroides*, *Akkermansia*, *Ruminococcaceae*, and *Prevotella*, along with an elevation in *Escherichia coli* and *Enterococcus*. These findings indicate a reduction in probiotic microorganisms and an increase in the abundance of pathogenic bacteria in patients with kidney failure [64]. These studies have shown that gut dysbiosis could be one of the contributors to increased systemic inflammation in CKD or ESRD patients. d-lactate, high-sensitive C-reactive protein, and interleukin-6 levels were substantially higher in these patients, indicating the onset of systemic inflammation [51,60,62]. Based on their research, Andersen et al. deduced that uremia is linked to intestinal dysbiosis, intestinal barrier dysfunction, and bacterial translocation, culminating in persistent systemic inflammation in CKD [47].

Not only can uremic status lead to gut dysbiosis, but gut dysbiosis can also affect kidney function. Two pathways through which gut dysbiosis can affect kidney function are metabolite and immune mediation [42,65]. Gut dysbiosis is linked to elevated production of intestinally derived metabolites, such as p-cresol, indole, and trimethylamine (TMA), which serve as precursors of uremic toxins [66,67]. In 2015, Xu and colleagues reported that the median plasma trimethylamine-N-oxide (TMAO) level was 30.33 μmol/L in CKD patients, nearly 15-fold higher compared to healthy controls [68]. Recent studies have revealed that alterations in TMAO levels are not only associated with uremia but also with dietary modifications in human trials [69,70], as well as changes in gut microbiota composition induced by metformin [71]. Uremia can lead to gut dysbiosis, characterized by an imbalance in gut bacteria, including an expansion of certain bacteria that possess uricase, p-cresyl-forming enzymes, and indole-forming enzymes [61,72]. Intestinal bacteria play a role in the generation of gut-derived uremic toxins, such as p-cresol sulfate (PCS) and indoxyl sulfate [73]. It is hypothesized that these gut-derived uremic toxins or their precursors may leak into the systemic circulation, contributing to kidney damage and increased cardiovascular risk [74]. Furthermore, in CKD, LPS from bacteria can enter the bloodstream [65], and there may also be instances of bacterial translocation [51]. The presence of LPS or bacteria in the circulation can induce systemic inflammation, potentially leading to kidney damage. Immune-mediated kidney damage arises from the activation of immune cells in the intestine due to dysbiosis [75]. The activated immune cells increase the production of cytokines [[42], [43], [44]] and soluble urokinase-type plasminogen activator receptor (suPAR) [[76], [77], [78]], resulting in kidney inflammation. Hayek et al. also found that an increased level of suPAR was independently linked to the development of CKD and a hastened decrease in estimated glomerular filtration rate (eGFR) [76].

Aside from CKD, it is worth noting that diet, alcohol, and various lifestyle-related factors, such as a Western diet, excessive alcohol consumption, and psychological stress, have also been associated with the development of gut dysbiosis [79]. In order to address gut dysbiosis and identify potential treatment targets for CKD, researchers have explored multiple interventions. These interventions include dietary modifications, fecal microbial transplantation, inhibitors targeting TMAO synthesis [80,81], as well as the supplementation of prebiotics or probiotics [82,83].

In brief, both age and CKD can result in gut dysbiosis and increased gut permeability, which are associated with activation of immune cells, increased production of cytokines and pro-inflammatory mediators, and local and systemic inflammation. The immune changes in aged-related gut dysbiosis and the chronic inflammation changes resulting from gut dysbiosis may also lead to CKD progression.

## The clinical results of inflamm-aging in chronic kidney disease (CKD) and end stage kidney disease (ESKD) patients

**Fig. 2:**
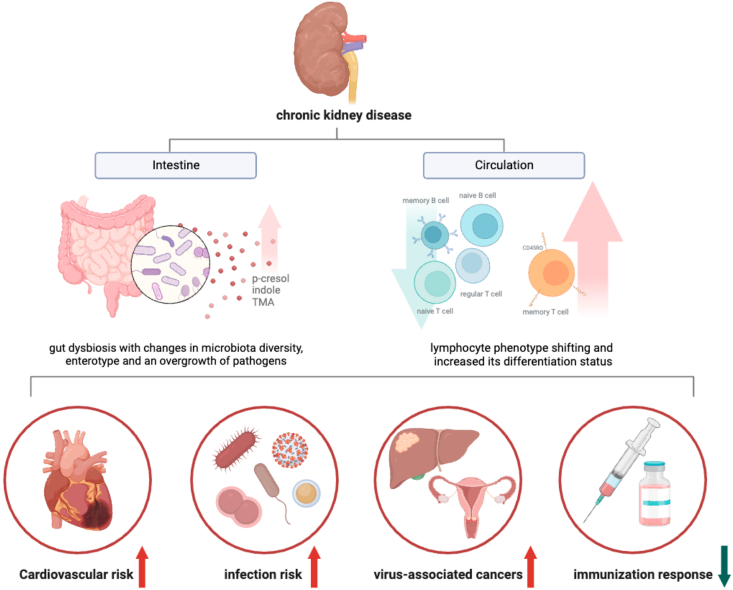
The clinical outcomes of inflamm-aging in patients with chronic kidney disease (CKD) and end stage kidney disease (ESKD).

The immune integrity of individuals with kidney function insufficiency is known to be compromised, and thus it is not surprising that patients with CKD and ESKD are highly susceptible to infections, exhibit reduced response to vaccination, and have an increased risk of cardiovascular events [Fig. 2] [3,4,39,84,85].

Advanced CKD is linked with an elevated risk of severe infections, such as urinary tract infections, lower respiratory tract infections, central nervous system infections, and sepsis [86]. The heightened risk of infection in advanced CKD is linked with subsequent negative outcomes. Based on their investigation of the Canadian Prospective Cohort (CanPREDDICT), Hassan and colleagues found that infectious episodes were independently associated with an elevated risk of cardiovascular ischemia, congestive heart failure, end-stage kidney disease, or mortality [87]. As kidney function decreases, the premature alterations in the immune system, such as reduced production of new T cells from the thymus, decreased relative telomere length of T cells [29], and a more differentiated memory T-cell compartment [25,26], make patients highly vulnerable to infections. This decline in kidney function creates a vicious cycle in CKD patients.

Moreover, vaccination seems to have little effect on breaking this vicious cycle. For instance, dialysis patients have been shown to have lower protective antibody titers and an inability to maintain adequate antibody titers over time after hepatitis B virus vaccination [84,88,89]. Therefore, the Centers for Disease Control and Prevention (CDC) recommends that CKD patients receive HBV vaccination early on in the course of kidney disease and receive higher doses and/or additional boosters to achieve adequate protection [88]. While annual influenza vaccination has been recommended for dialysis patients, Remschmidt et al.'s meta-analysis indicates that the protective benefits of influenza vaccination in individuals with ESKD are restricted and of poor quality [85].

During the COVID-19 pandemic, CKD has been identified as a key risk factor for mortality [[90], [91], [92]]. Gansevoort and colleagues reported a graded association between the level of kidney dysfunction and the risk of COVID-19 mortality [91]. Based on the OpenSAFELY project, which examined 17 million patients, dialysis, organ transplant, and stage 4 and 5 CKD were three of the four comorbidities associated with the highest mortality risk from COVID-19 [90]. The higher risk of virus infection might be associated with the relative lowering level of T_FH1_ in CKD patients [32], which plays a pivotal role in response to viral infection [93,94]. Furthermore, studies have demonstrated that the immunogenicity rate after vaccination is lower in CKD and ESKD patients [36,95,96]. Besides, a state of chronic systemic inflammation also increases morbidity and mortality in kidney insufficiency patients [97,98]. In brief, the phenomenon of inflamm-aging in individuals with kidney insufficiency renders this population susceptible to diminished immunogenicity following vaccination, while the presence of chronic inflammation and inflamm-aging renders CKD patients prone to heightened morbidity and mortality throughout the COVID-19 pandemic.

### Conclusion

CKD and ESKD patients face an immunosenescence status nearly 20 years earlier compared to age-matched healthy individuals [1]. The accumulation of uremic toxins influence the innate immunity via upregulation of chemokine/chemokine and ROS production [24], impaired phagocytosis, and impaired stimulation of T cells [20]. In adaptive immune system, uremic status shifts lymphocytes towards senescent and exhausted phenotypes, lowers thymic T cell output, and decreases the relative telomere length of T cells [28], weakening the adaptive immune system in kidney insufficiency. In addition, Uremia is also associated with gut dysbiosis, which results in alterations in the diversity and abundance of gut microbiota. These changes further enhance the production of precursors of uremic toxins, ultimately exacerbating both local and systemic inflammation. Consequently, the accumulation of gut-derived uremic toxins contributes to an accelerated decline in kidney function [[64], [65], [66], [67],^76^]. The immunosenescence, which results in impaired vaccine responses, increased susceptibility to infection, autoimmunity, and cancer, as well as increased cardiovascular risk, may explain the fragility and premature mortality in CKD and ESKD patients [3,4,39,84,85].

## Funding

This review article was supported by grants from the 10.13039/100012553Chang Gung Memorial Hospital (CMRPG3I0371 and CMRPG3I0372).

## Conflicts of interest

The authors declare no conflicts of interest.
